# Census Tract Patterns and Contextual Social Determinants of Health Associated With COVID-19 in a Hispanic Population From South Texas: A Spatiotemporal Perspective

**DOI:** 10.2196/29205

**Published:** 2021-08-05

**Authors:** Cici Bauer, Kehe Zhang, Miryoung Lee, Susan Fisher-Hoch, Esmeralda Guajardo, Joseph McCormick, Isela de la Cerda, Maria E Fernandez, Belinda Reininger

**Affiliations:** 1 Department of Biostatistics and Data Science School of Public Health The University of Texas Health Science Center at Houston Houston, TX United States; 2 Department of Epidemiology, Human Genetics and Environmental Science School of Public Health The University of Texas Health Science Center at Houston Brownsville, TX United States; 3 Cameron County Public Health San Benito, TX United States; 4 Department of Health Promotion and Behavior Sciences School of Public Health The University of Texas Health Science Center at Houston Houston, TX United States; 5 Department of Health Promotion and Behavior Sciences School of Public Health The University of Texas Health Science Center at Houston Brownsville, TX United States

**Keywords:** COVID-19, spatial pattern, social determinants of health, Bayesian, underserved population, health inequity

## Abstract

**Background:**

Previous studies have shown that various social determinants of health (SDOH) may have contributed to the disparities in COVID-19 incidence and mortality among minorities and underserved populations at the county or zip code level.

**Objective:**

This analysis was carried out at a granular spatial resolution of census tracts to explore the spatial patterns and contextual SDOH associated with COVID-19 incidence from a Hispanic population mostly consisting of a Mexican American population living in Cameron County, Texas on the border of the United States and Mexico. We performed age-stratified analysis to identify different contributing SDOH and quantify their effects by age groups.

**Methods:**

We included all reported COVID-19–positive cases confirmed by reverse transcription–polymerase chain reaction testing between March 18 (first case reported) and December 16, 2020, in Cameron County, Texas. Confirmed COVID-19 cases were aggregated to weekly counts by census tracts. We adopted a Bayesian spatiotemporal negative binomial model to investigate the COVID-19 incidence rate in relation to census tract demographics and SDOH obtained from the American Community Survey. Moreover, we investigated the impact of local mitigation policy on COVID-19 by creating the binary variable “shelter-in-place.” The analysis was performed on all COVID-19–confirmed cases and age-stratified subgroups.

**Results:**

Our analysis revealed that the relative incidence risk (RR) of COVID-19 was higher among census tracts with a higher percentage of single-parent households (RR=1.016, 95% posterior credible intervals [CIs] 1.005, 1.027) and a higher percentage of the population with limited English proficiency (RR=1.015, 95% CI 1.003, 1.028). Lower RR was associated with lower income (RR=0.972, 95% CI 0.953, 0.993) and the percentage of the population younger than 18 years (RR=0.976, 95% CI 0.959, 0.993). The most significant association was related to the “shelter-in-place” variable, where the incidence risk of COVID-19 was reduced by over 50%, comparing the time periods when the policy was present versus absent (RR=0.506, 95% CI 0.454, 0.563). Moreover, age-stratified analyses identified different significant contributing factors and a varying magnitude of the “shelter-in-place” effect.

**Conclusions:**

In our study, SDOH including social environment and local emergency measures were identified in relation to COVID-19 incidence risk at the census tract level in a highly disadvantaged population with limited health care access and a high prevalence of chronic conditions. Results from our analysis provide key knowledge to design efficient testing strategies and assist local public health departments in COVID-19 control, mitigation, and implementation of vaccine strategies.

## Introduction

COVID-19, which comes from SARS-CoV-2, has caused death, health care system stress, and global economic instability. In the United States, it also has disproportionately affected minority and underserved populations, where COVID-19 infection and fatality rates are significantly higher among African American and Hispanic populations [1-3]. Previous studies have shown various social determinants of health (SDOH) that may explain the disparity in COVID-19 incidence and mortality in ethnic and racial minorities [1,4,5].

The differential impact of COVID-19 on minorities and other groups facing health inequities has been described and underscores a critical need to target these underserved groups. However, the majority of these studies in the United States used aggregated county-level data from the COVID Tracking Project [6]. The geographical scale of the US county often lacks granularity to reveal the local spatial pattern and detect local hot spots (ie, areas with excessive infection rates). Moreover, the high variability of SDOH within a county population was not able to accurately examine the impact of SDOH on COVID-19 disparities in populations [5]. Studies that investigate the SDOH and COVID-19 incidence and mortality at a geographical scale smaller than the US county are limited [7,8]. The lack of studies on a granular spatial scale is largely due to insufficiently detailed COVID-19 surveillance data, particularly data that are publicly available.

In this study, we investigated the contextual SDOH and their potential association with COVID-19 incidence at the census tract level. The study population consists of a Hispanic population with mostly Mexican American people living in South Texas on the US-Mexico border. The Mexican American population are the largest and fastest-growing Hispanic subgroup in the United States and among those with low socioeconomic status compared to other ethnic groups in the nation [9]. The population in our study has high prevalence of obesity and diabetes [10]; both pre-existing conditions increase the risk of severe COVID-19 outcomes [11]. Our analysis provided a look at the SDOH at sufficient spatial granularity to detect local trends and hot spots for COVID-19 monitoring and control. Results from our study have informed the intervention strategies to increase COVID-19 testing uptake in underserved populations and the design of interventions and targeted vaccination programs.

## Methods

### Study Population

**Figure 1:**
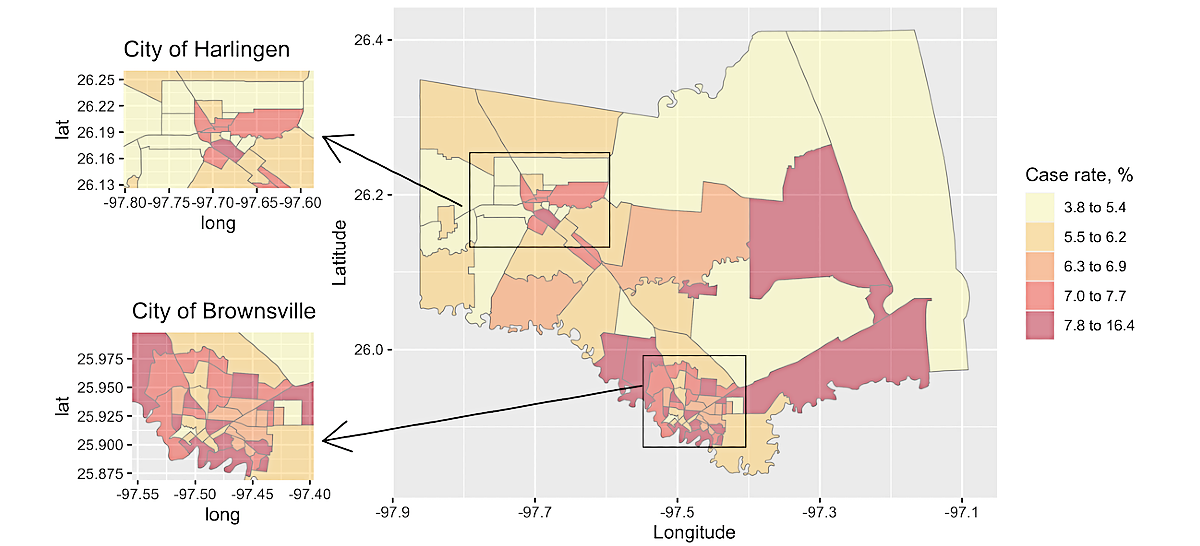
Choropleth map presenting the cumulative COVID-19 infection rate by census tract between March 18, 2020, and December 16, 2020, in Cameron County, TX. The two largest cities are the city of Brownsville on the border of the United States and Mexico (bottom left panel) and the city of Harlingen (top left panel).

Our study population is from Cameron County, Texas with a current population of 423,163 and over 90% Hispanics [12], where the vast majority were Mexican-Hispanic [13]. Most Cameron County residents are uninsured (~29%) and live below the poverty line (~33%) [12]; additional research based on a well-documented cohort from this region estimated that around 52% of the population does not have any private or public health insurance coverage [10,13]. This population, similar to many others living in the South Texas region, also has a high prevalence of type 2 diabetes (over 27%) and obesity (over 50%) [10,13,14]. In our analysis, we included a total of 84 census tracts within Cameron County, as shown in Figure 1. The two largest cities in Cameron County are the City of Brownsville (population 183,677) on the US-Mexico border and the City of Harlingen (population 65,074) 20 miles north of Brownsville, together comprising 59% of the county population.

### COVID-19 Reported Cases

The first confirmed COVID-19 case in Cameron County, Texas was reported on March 18, 2020. By December 16, a total of 28,111 cases had been reported. The cumulative case rate, calculated as the number of positive cases per 100 people, was 1.93% in May 2020 and increased to 6.64% by December 2020, when the cumulative case rate of the general US population in December 2020 was 5.16% [6]. The case-fatality rate in Cameron County was 4% compared to 1.98% in Texas by December.

To facilitate local COVID-19 control and mitigation, Cameron County Public Health Department, the City of Brownsville, and the University of Texas School of Public Health (UTHealth) formed a collaborative group soon after the first COVID-19 case was reported in March 2020. Cameron County Public Health Department maintains a local database of reported and confirmed COVID-19 cases, which were concurrently reported to the Texas Department of State Health Services through the National Electronics Disease Surveillance System. Researchers from UTHealth were given access to the local database and provided data management and analytical support to investigate the trends and risk factors associated with COVID-19 spread. This study was approved by the UTHealth Committee for the Protection of Human Subjects (HSC-SPH-20-1372) and the Data User Agreement between the UTHealth and Cameron County.

**Figure 2:**
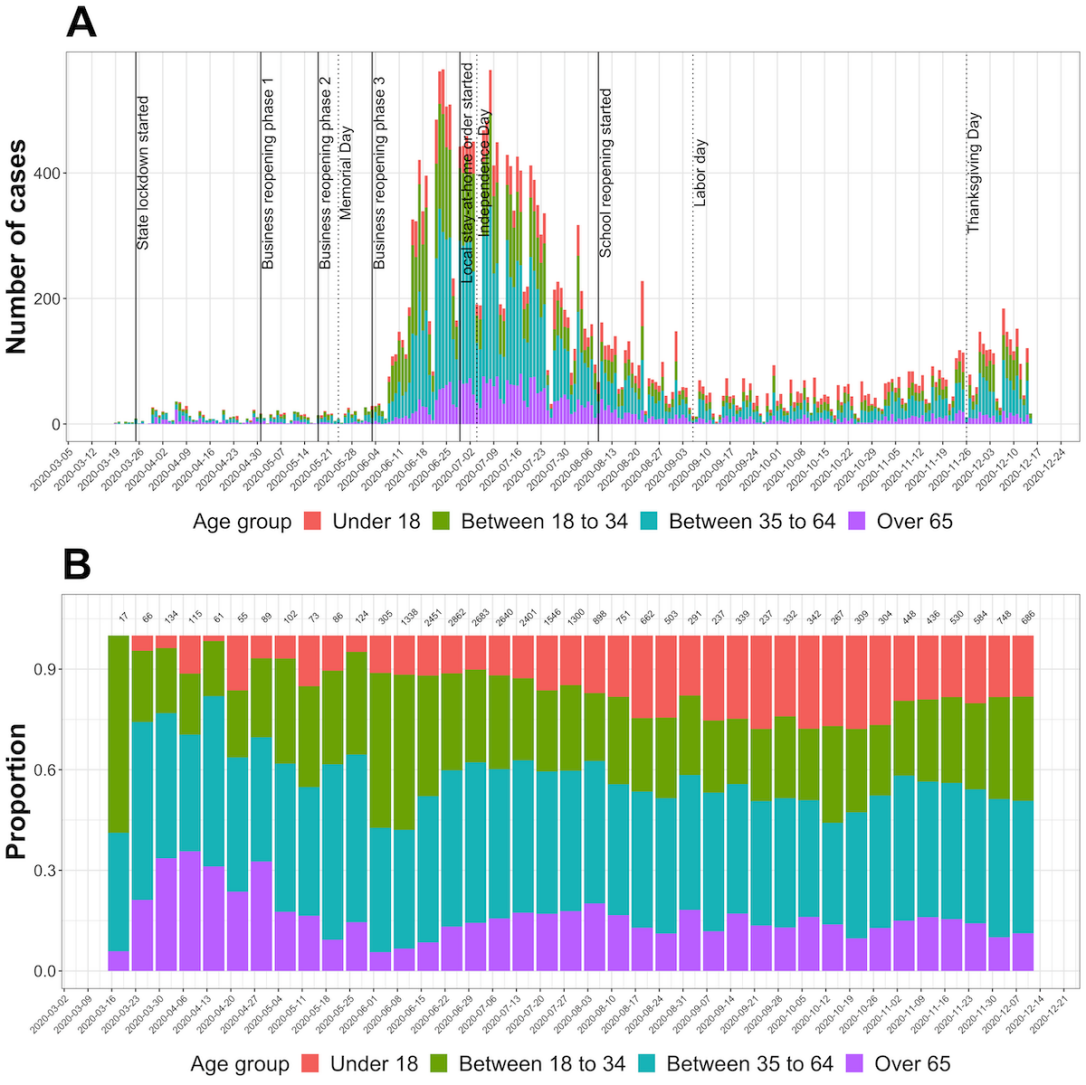
Temporal pattern of COVID-19–confirmed cases by age groups in Cameron County, Texas between March 18 and December 16, 2020. Panel (A) presents the weekly counts by age groups, along with the event timeline of the state or local COVID-19 mitigation and control policies (solid line) and holidays (dashed line). Panel (B) presents the relative proportions of the weekly cases by age groups, where total weekly counts are shown at the top margin.

During the initial stage of the COVID-19 pandemic, individual-level information associated with each reported case included age, gender, ethnicity, race, residential address, and specimen collection date. Over time, additional variables were added to the database such as testing type, testing facility, case definition (ie, confirmed or probable), type of exposure, date of recovery, date of death, comorbidities, hospitalization, symptoms, and symptoms onset date. In this analysis, we included all reported COVID-19–positive cases confirmed by reverse transcription–polymerase chain reaction testing based on a sample collection date between March 18 and December 16, 2020, and with a reported residential address within Cameron County. We developed a geocoding algorithm that extracted residential address information and then obtained the corresponding census tract information using the Google application programming interface (API) and the Census Bureau API in R (R Foundation for Statistical Computing) [15,16]. Among the total of 28,111 cases, we were able to geocode 27,733 cases and obtained their census tract information. Of these, 27,731 cases had information on sex, with 14,903 (53.8%) females and 12,824 (46.2%) males. Of the 27,726 cases (missing 1.37%) with age information, 15% (n=4148) were younger than 18 years, 28% (n=7770) were between age 18 to 34 years, 42.7% (n=11,843) were between age 35 to 64 years, and 14.2% (n=3965) were 65 years and older. The age strata range was chosen based on the US Centers for Disease Control and Prevention (CDC) COVID-19 case reporting [6], with some age groups collapsed due to small case numbers. Figure 2 presented weekly confirmed cases stratified by these age groups during the study time.

### Demographic and Social Determinants of Health

Census tract demographic and SDOH variables for Cameron County were obtained from 2013 to 2018 US Census Bureau American Community Survey (ACS) 5-year estimates. These variables included total population, unemployment (%), racial minority (%), poverty level (% living under poverty), education level (% with no high school diploma), income (per capita income in dollars), insurance (% of population uninsured), living conditions (% renters and % living in crowded housing), and transportation (% without vehicles). We also created a population density variable for the census tracts, calculated as the population size per kilometer squared (km^2^), ranging from 17 to 1360 per km^2^. We observed substantial spatial variation of these demographic and SDOH within the Cameron County (Multimedia Appendix 1).

### Shelter-in-Place Indicator Variable

To evaluate the impact of local policy on COVID-19, we created a binary indicator variable with value 1 for time periods when a state or local stay-at-home order was in place, and value 0 otherwise. Mandatory policies of facial coverings, curfew, limitations on gatherings, or beach access closure were present during the shelter-in-place periods [17]. The time period between March 26 and May 1, 2020, corresponded to the presence of the state-level lockdown, at the end of which the phased business reopening began. The local stay-at-home order started from July 1, 2020, and became less restrictive after schools reopened in mid-August. The event timeline of the policy and holidays is shown in Figure 2 (panel A).

### Statistical Analysis

Due to potential reporting lag, we aggregated the number of COVID-19–confirmed cases to weekly counts by census tract. We considered the following Bayesian spatiotemporal model [18,19]. Let *Y_it_* denote the number of confirmed cases from census tract *i* and week *t*; we assumed a negative binomial distribution with incidence risk *μ_it_* (ie, *Y_it_*|*μ_it_* ~ *NB*(*N_i_μ_it_*), with *N_it_* the population size as the offset. The incidence risk was *μ_it_* then modeled as follows:

log (μ_it_) = α + x'_i_β + s_t_γ + φ_i_ + δ_it_

where *α* was the overall intercept, *x_i_* was the vector of census tract covariates (eg, unemployment and crowded housing) with the associated coefficient vector *β*. Covariate *s_t_* was the binary policy-in-place indicator previously described. To account for the tract-level spatial dependency, we included a spatial random effect *φ_i_* using the intrinsic conditional autogressive model [20]. The spatiotemporal interaction term *δ_it_* captured the unexplained residuals and was assumed an independent and identically distributed normal distribution with variance 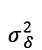. We reported the relative risk (RR) associated with each covariate, which was calculated as the exponentiated coefficient, along with its 95% posterior credible intervals (CIs). We performed this model on the total COVID-19 cases and then on age subgroups of younger than 18 years, between ages 18 and 34 years, between ages 35 and 64 years, and older than 65 years. All analyses were performed in R [21] and R package INLA [22].

## Results

Compared to the US general population, Cameron County has a higher proportion of people who are uninsured (29.1% vs 9.4%), living under poverty (29.6% vs 11.5%), less educated (36.2% with no high school diploma vs 13%), and with worse living conditions (11.8% with crowded housing vs 3.4%). Cameron County is also 90.6% Hispanic, in contrast to 38.3% nationally, and 75% of the population with Spanish as the primary language and 28% having limited English proficiency (Table 1).

Figure 2 presents the temporal patterns of COVID-19–confirmed cases, in total numbers and by proportion, between March and December 2020. We observed a clear increase in new cases starting in June, that gradually decreased through the end of August. At the beginning of the pandemic in March and April, most cases were from the older population; more cases emerged from the younger population as the pandemic progressed to the summer. Cases among those 18 years or younger substantially increased from June and peaked in September. Unlike the three waves seen in the US general population, we only observed one prominent wave during the summer, with a smaller second wave after the Thanksgiving holiday.

**Figure 3:**
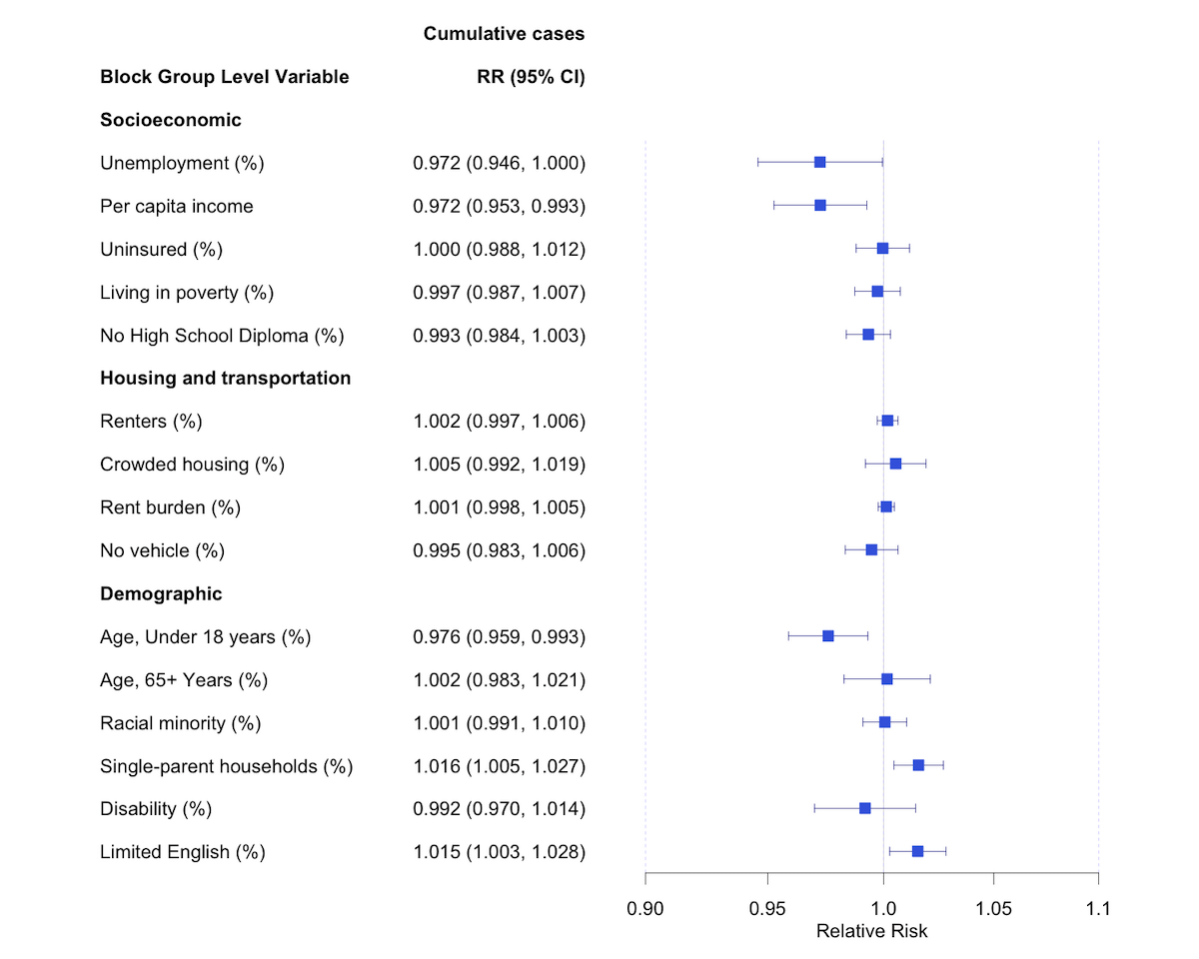
Estimated RRs and posterior 95% credible intervals associated with census tract social determinants of health. Estimates are obtained from fitting a Bayesian spatiotemporal negative binomial on all COVID-19 confirmed cases from Cameron County, TX between March 18, 2020, and December 16, 2020. RR: relative risk.

**Figure 4:**
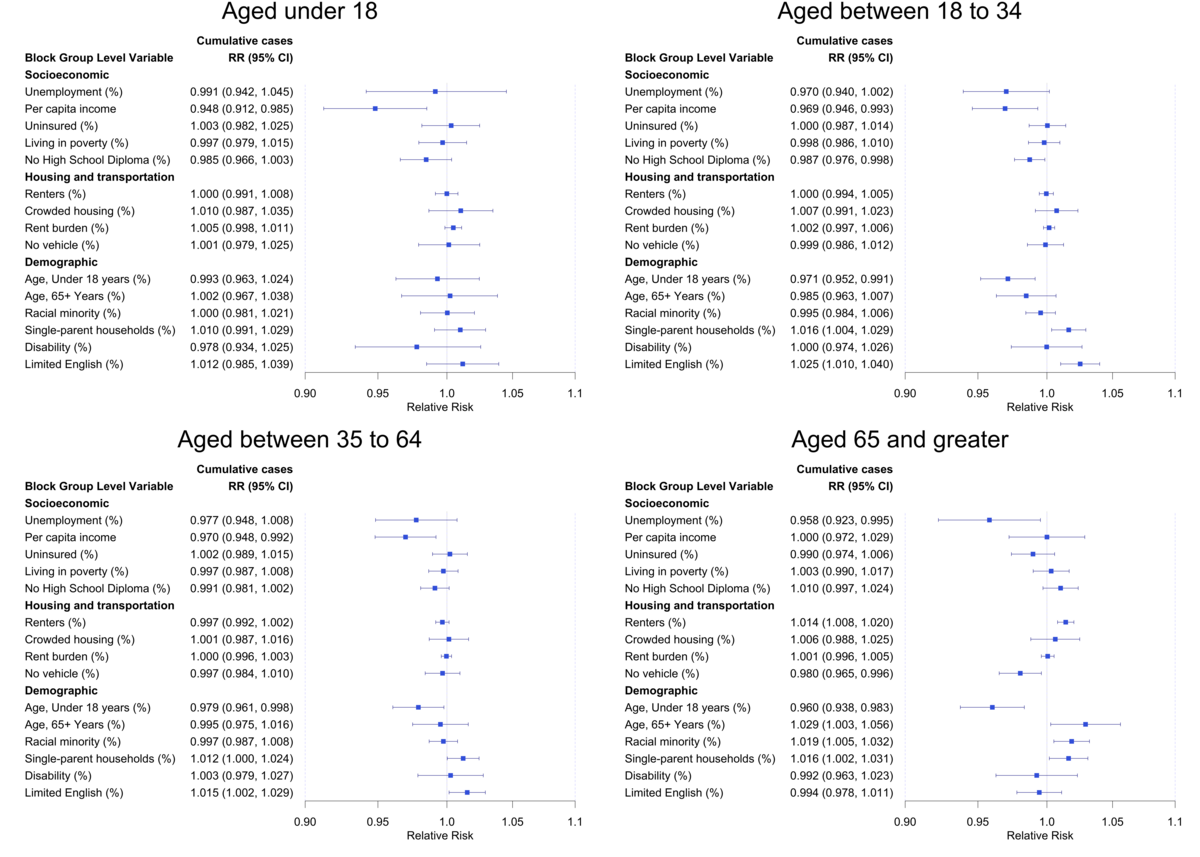
Estimated RRs and posterior 95% credible intervals associated with census tract social determinants of health. Estimates are obtained from fitting a Bayesian spatiotemporal negative binomial model and stratified by age groups, using COVID-19–confirmed cases from Cameron County, TX between March 18, 2020, and December 16, 2020. RR: relative risk.

We fit the Bayesian spatiotemporal negative binomial model previously described to all COVID-19–confirmed cases and then to four age-stratified subgroups (age younger than18 years, 19-35 years, 36-64 years, 65 years and older), and the results are presented in Figures 3 and 4. Of the various demographic and SDOH variables included, the RR of COVID-19 incidence was higher among census tracts with a higher percentage of single-parent households (RR=1.016, 95% CI 1.005, 1.027) and a higher percentage of the population with limited English proficiency (RR=1.015, 95% CI 1.003, 1.028). Lower income was associated with a reduced risk of COVID-19 (RR=0.972, 95% CI 0.953, 0.993) as was the percentage of the population younger than 18 years (RR=0.976, 95% CI 0.959, 0.993). The most striking association was the *shelter-in-place* variable, where the RR of COVID-19 incidence was 0.506 (95% CI 0.454, 0.563) when comparing policy present versus policy absent. This suggests the risk of COVID-19 was reduced by almost 50% when the *shelter-in-place* policy was present.

Age-stratified analyses identified different significant SDOH for each group, and results are presented in Figure 4. For the age group 19 to 34 years, the estimated RR associated with higher percentage of limited English proficiency was 1.025 (95% CI 1.010, 1.040), a higher risk compared to that of the overall population (RR=1.015, 95% CI 1.003, 1.028). Reduced COVID-19 risk was associated with census tracts with higher percentage of no high school education (RR=0.987, 95% CI 0.976, 0.998). For the age group 65 years and older, the percentages of renters and racial minority (ie, percentage of non-Hispanic White) were additional SDOH significantly associated with increased risk of COVID-19 (RR=1.014, 95% CI 1.008, 1.020 and RR=1.018, 95% CI 1.005, 1.032, respectively). The complete results are presented in Multimedia Appendix 2.

The COVID-19 incidence risk was consistently and substantially lower during the time when the “shelter-in-place” policy was present. The effect was the most remarkable for the age group 19 to 35 years, where the risk was reduced by almost 60% when the policy was in place (RR=0.378, 95% CI 0.335, 0.425). For the age group 35 to 65 years, the risk was reduced by almost 50% (RR=0.475, 95% CI 0.424, 0.532). COVID-19 risk reduction was attenuated for the age group 65 years and older (RR=0.690, 95% CI 0.599, 0.793) and the smallest for the age group 18 years or younger (RR=0.767, 95% CI 0.667, 0.881).

**Table 1:** Summary statistics of the census tract demographics and social determinants of health in Cameron County, Texas and the whole United States. Data were obtained from American Community Survey 2013-2018 5-year estimates.

Variable		Cameron (n=84)	US (n=73,056)
**Younger than 18 years (%)**			
	Mean (CV^a^ %)	30.2 (18.9)	22.1 (30.1)
	Median (Q1, Q3^b^)	31.2 (27.5, 33.7)	22.2 (18.5, 26.0)
**Older than 65 years (%)**			
	Mean (CV %)	14.0 (38.3)	16.0 (50.2)
	Median (Q1, Q3)	13.2 (10.4, 16.9)	15.2 (11.0, 19.6)
**Racial minority (%)**			
	Mean (CV %)	90.6 (12.1)	38.3 (78.3)
	Median (Q1, Q3)	94.7 (86.8, 97.1)	29.7 (12.5, 60.8)
**Single-parent household (%)**			
	Mean (CV %)	14.6 (39.0)	9.3 (69.2)
	Median (Q1, Q3)	14.1 (10.1, 18.8)	7.9 (4.8, 12.2)
**Disability (%)**			
	Mean (CV %)	13.5 (30.1)	13.4 (44.0)
	Median (Q1, Q3)	13.7 (10.4, 16.2)	12.5 (9.2, 16.6)
**Limited English (%)**			
	Mean (CV %)	27.7 (35.4)	8.0 (135.4)
	Median (Q1, Q3)	27.6 (20.4, 35.7)	3.5 (1.1, 10.1)
**Unemployed (%)**			
	Mean (CV %)	3.9 (51.5)	3.9 (68.1)
	Median (Q1, Q3)	3.5 (2.4, 5.1)	3.3 (2.1, 4.9)
**No high school diploma (%)**			
	Mean (CV %)	36.2 (39.1)	13.0 (81.2)
	Median (Q1, Q3)	35.7 (24.3, 48.6)	10.1 (5.4, 17.6)
**Per capita income (US $)**			
	Mean (CV %)	16,100 (42.5)	32,300 (52.1)
	Median (Q1, Q3)	14,000 (11,300, 19,500)	28,600 (21,700, 38,200)
**Living poverty (%)**			
	Mean (CV %)	29.6 (38.6)	11.5 (93.1)
	Median (Q1, Q3)	28.9 (21.0, 37.1)	8.2 (3.8, 15.9)
**Uninsured (%)**			
	Mean (CV %)	29.1 (29.1)	9.4 (75.7)
	Median (Q1, Q3)	29.1 (23.3, 34.3)	7.6 (4.2, 12.6)
**Crowded housing (%)**			
	Mean (CV %)	11.8 (52.9)	3.6 (146.1)
	Median (Q1, Q3)	11.2 (7.5, 15.0)	1.9 (0.6, 4.4)
**Renters (%)**			
	Mean (CV %)	36.9 (46.5)	36.8 (62.3)
	Median (Q1, Q3)	34.5 (24.9, 44.9)	31.7 (18.6, 51.4)
**Rent burden (%)**			
	Mean (CV %)	55.8 (26.4)	48.2 (33.4)
	Median (Q1, Q3)	55.0 (48.9, 65.0)	48.6 (38.0, 59.0)
**No vehicle (%)**			
	Mean (CV %)	8.6 (81.1)	9.4 (130.4)
	Median (Q1, Q3)	7.40 (3.5, 11.0)	5.3 (2.5, 11.0)

^a^CV: coefficient of variation.

^b^Q1, Q3: first quartile, third quartile.

## Discussion

Using reported and confirmed COVID-19 cases from the Cameron County Public Health Department, we identified SDOH that were associated with COVID-19 incidence risk at the census tract level for the overall population and age subgroups. Risk of COVID-19 incidence was statistically significantly higher among areas with higher percentages of single-parent households and limited English-speaking proficiency but lower among areas with younger populations and lower income. The protective effects of lower income (for all cases) and lower education (for the age group 19-34 years) were difficult to decipher. On one hand, people living in low employment areas during the pandemic may have reduced contact with those infected and hence less likely to get infected. On the other hand, people with *essential jobs* (eg, food services) also tend to live in low income and low education areas. They may not be able to shelter at home like those in other jobs and hence have a higher risk of getting infected. For example, a previous study from Orange County, California showed an increase in COVID-19 cases in Hispanic and Latinx populations who lived in low-income census tracts and had low education attainment [23]. We were not able to further investigate the association with the census tract unemployment rate due to the lack of employment data at the census tract level during the pandemic. Other SDOH variables and social vulnerability indices such as those provided by the CDC [24] were not included in this analysis since they are typically constructed using the ACS variables we included in this analysis or tend to be highly associated with those included. Our result on the *shelter-in-place* policy agreed with previous studies where stay-at-home orders were effective in decreasing the confirmed case growth rate [25], and cumulative COVID-19 cases fell by about 50% following 3 weeks of a *shelter-in-place* order [26], but the effects vary in magnitude by age subgroups.

Our study has some limitations. First, our analysis only included the reported and confirmed cases, and hence missed those that were unreported or undiagnosed. Second, we were not able to evaluate the individual contribution of each different mitigation plan on reducing COVID-19 incidence risk. Third, we could not include the pre-existing conditions such as diabetes and obesity prevalence in our analysis, which were shown to impact COVID-19 severity but were unclear on infection. Finally, and probably the most important one, is that we were not able to include the overall testing data due to the lack of complete and accurate testing data by census tract level in the study region. Accurately capturing the COVID-19 pandemic requires an enhanced surveillance database, where ideally testing and infection data can be linked at the individual level. We hope in our future endeavor to assist the county and city public health departments to construct a comprehensive surveillance database as such to provide real-time monitoring and early detection of future COVID-19 outbreaks.

The population we focus on in this analysis is one of the poorest in the United States, frequently uninsured, and with limited access to COVID-19 testing throughout the pandemic. Using a Bayesian spatiotemporal binomial model, we investigated the association of SDOH and COVID-19 *shelter-in-place* policies with confirmed COVID-19 cases. Though there has been a surge of studies investigating the association of SDOH and COVID-19–related health outcomes since the pandemic started, most of them focused on the county-level analysis [27-30]. This spatial unit may lack the granularity to detect local hotpots and, subsequently, is inadequate to inform the local public health officials for mitigation control and planning. To our knowledge, our study is the first conducted at a granular spatial scale of census tracts and on a highly disadvantaged Hispanic population with limited health care access and a high chronic health risk including diabetes and obesity. The analysis also provided key information in guiding the intervention strategies to increase the testing uptake in the underserved population. For example, we are currently using this methodology as part of the Rapid Access to Diagnostics for Underserved Populations program that aims to increase knowledge about and access to testing in high-risk communities. The information generated from this study and the application of this methodology is informing both the development of targeted intervention strategies and the deployment of services to these areas.

## Appendix

Multimedia Appendix 1 Maps of demographic and social determinants of health variables at census tract level in Cameron County, TX. Data were obtained from American Community Survey 2013-2018 5-year estimates. At the tract level, the average percentage of Hispanics is 89.4% (SD 13%), much higher compared to the national average of 16.4%.

Multimedia Appendix 2 Census tract level estimated relative risks associated with social determinants of health variables, with posterior 95% credible intervals in parentheses. The relative risk (RR) estimates were obtained from fitting a Bayesian negative binomial regression model, with spatial and spatiotemporal random effects. RRs with statistically significant results are shown in bold. COVID-19 case data between March 19, 2020, and December 16, 2020, from Cameron County, TX was used in reporting the results.

## Abbreviations

ACS: American Community Survey
API: application programming interface
CDC: Centers for Disease Control and Prevention
CI: credible interval
RR: relative risk
SDOH: social determinants of health
UTHealth: University of Texas School of Public Health
