# Correction: Census Tract Patterns and Contextual Social Determinants of Health Associated With COVID-19 in a Hispanic Population From South Texas: A Spatiotemporal Perspective

**DOI:** 10.2196/32870

**Published:** 2021-08-18

**Authors:** Cici Bauer, Kehe Zhang, Miryoung Lee, Susan Fisher-Hoch, Esmeralda Guajardo, Joseph McCormick, Isela de la Cerda, Maria E Fernandez, Belinda Reininger

**Affiliations:** 1 Department of Biostatistics and Data Science School of Public Health The University of Texas Health Science Center at Houston Houston, TX United States; 2 Department of Epidemiology, Human Genetics and Environmental Science School of Public Health The University of Texas Health Science Center at Houston Brownsville, TX United States; 3 Cameron County Public Health San Benito, TX United States; 4 Department of Health Promotion and Behavior Sciences School of Public Health The University of Texas Health Science Center at Houston Houston, TX United States; 5 Department of Health Promotion and Behavior Sciences School of Public Health The University of Texas Health Science Center at Houston Brownsville, TX United States

In “Census Tract Patterns and Contextual Social Determinants of Health Associated With COVID-19 in a Hispanic Population From South Texas: A Spatiotemporal Perspective” (JMIR Public Health Surveill 2021;7(8):e29205), one error was noted.

Due to a system error, the name of one author, Joseph McCormick, was replaced with the name of another author on the paper, Isela de la Cerda. In the originally published paper, the order of authors was listed as follows:

Cici Bauer, Kehe Zhang, Miryoung Lee, Susan Fisher-Hoch, Esmeralda Guajardo, Isela de la Cerda, Isela de la Cerda, Maria E Fernandez, Belinda Reininger

This has been corrected to:

Cici Bauer, Kehe Zhang, Miryoung Lee, Susan Fisher-Hoch, Esmeralda Guajardo, Joseph McCormick, Isela de la Cerda, Maria E Fernandez, Belinda Reininger

In the originally published paper, the ORCID of author Isela de la Cerda was incorrectly published as follows:

0000-0002-5844-8102

This has been corrected to:

0000-0003-3625-8954

The correction will appear in the online version of the paper on the JMIR Publications website on August 18, 2021, together with the publication of this correction notice. Because this was made after submission to PubMed, PubMed Central, and other full-text repositories, the corrected article has also been resubmitted to those repositories.

