# Supplementary figures and images for: Census Tract Patterns and Contextual Social Determinants of Health Associated With COVID-19 in a Hispanic Population From South Texas: A Spatiotemporal Perspective
Source: JMIR Public Health Surveill. 2021 Aug 5;7(8):e29205. doi: 10.2196/29205 (PMC8354426; doi:10.2196/29205)

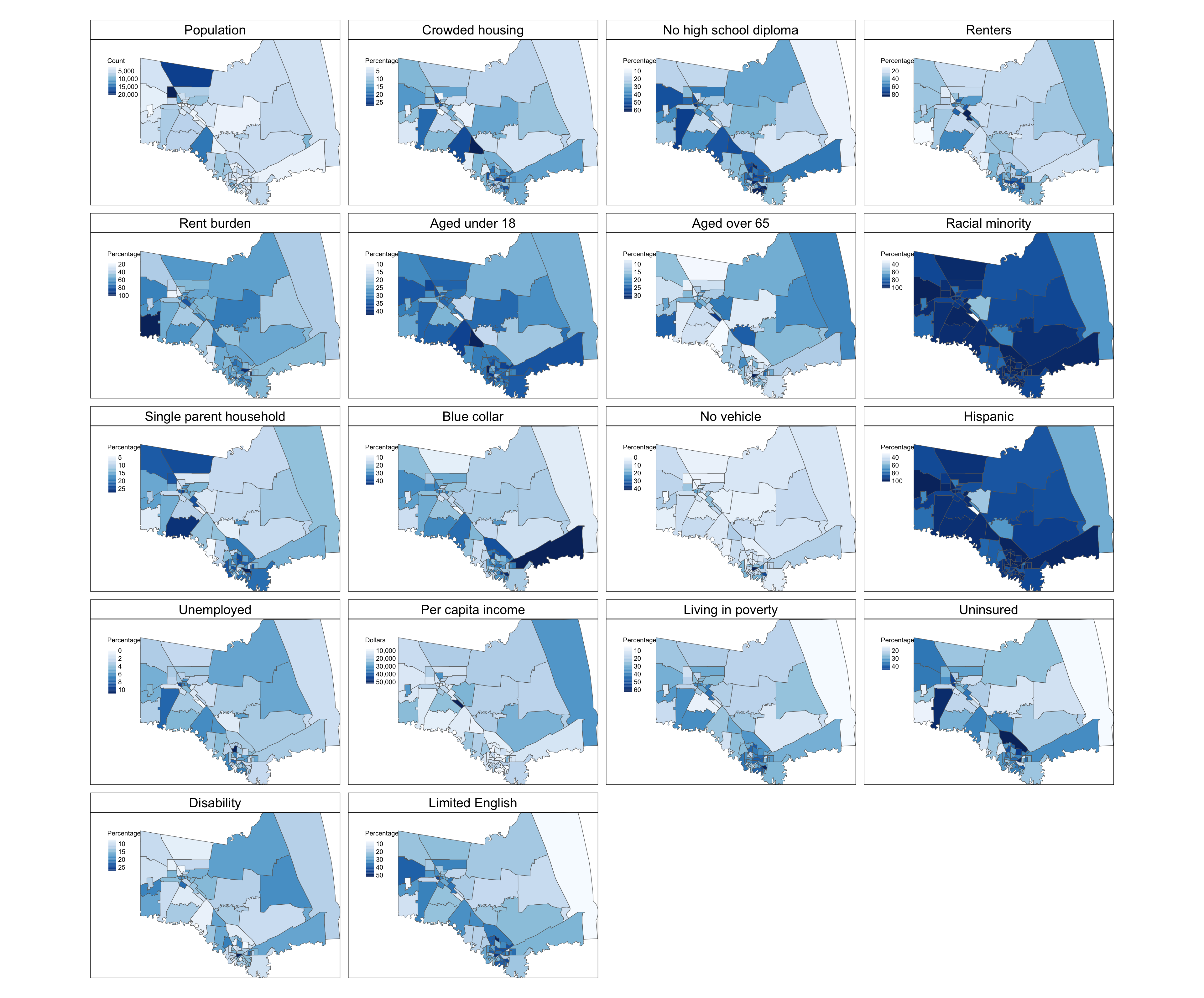

Supplement: Multimedia Appendix 1 [file publichealth_v7i8e29205_app1.png]
