# Supplementary material for: Census Tract Patterns and Contextual Social Determinants of Health Associated With COVID-19 in a Hispanic Population From South Texas: A Spatiotemporal Perspective
Source: JMIR Public Health Surveill. 2021 Aug 5;7(8):e29205. doi: 10.2196/29205 (PMC8354426; doi:10.2196/29205)
Supplement: Multimedia Appendix 2 [file publichealth_v7i8e29205_app2.docx]

**Multimedia Appendix**

**Census-tract level estimated relative risk (RR) associated with SDOH variables, with posterior 95% credible intervals in parentheses. The RR estimates were obtained from fitting a Bayesian Negative Binomial regression model, with spatial and spatiotemporal random effects. RR with statistically significant results are shown in bold. COVID-19 case data between March 19^th^, 2020 and December 16^th^, 2020 from Cameron County, TX was used in reporting the results.**

|  | Overall | Age under 18 | Age 19 - 34 | Age 35 - 65 | Age over 65 |
| --- | --- | --- | --- | --- | --- |
| Total cases | 27352 | 4117 | 7661 | 11685 | 3895 |
| Shelter in place | **0.506 (0.454, 0.563)** | **0.767 (0.667, 0.881)** | **0.378 (0.335, 0.425)** | **0.475 (0.424, 0.532)** | **0.690 (0.599, 0.793)** |
| Population Density (per km^2^) | 1.000 (1.000, 1.000) | 1.000 (1.000, 1.000) | 1.000 (1.000, 1.000) | 1.000 (1.000, 1.000) | 1.000 (1.000, 1.000) |
| Unemployment (%) | **0.972 (0.946, 1.000)** | 0.992 (0.942, 1.046) | 0.970 (0.940, 1.002) | 0.977 (0.948, 1.008) | **0.958 (0.923, 0.995)** |
| Per capita income ($1K) | **0.972 (0.953, 0.993)** | **0.948 (0.912, 0.985)** | **0.969 (0.946, 0.993)** | **0.970 (0.948, 0.992)** | 1.000 (0.972, 1.028) |
| Uninsured (%) | 1.000 (0.988, 1.012) | 1.003 (0.981, 1.025) | 1.000 (0.987, 1.014) | 1.002 (0.989, 1.015) | 0.990 (0.974, 1.006) |
| Poverty (%) | 0.997 (0.987, 1.007) | 0.997 (0.979, 1.015) | 0.998 (0.986, 1.010) | 0.997 (0.987, 1.008) | 1.003 (0.990, 1.017) |
| Renters (%) | 1.002 (0.997, 1.006) | 1.000 (0.991, 1.008) | 1.000 (0.994, 1.005) | 0.997 (0.992, 1.002) | **1.014 (1.008, 1.020)** |
| Crowded housing (%) | 1.005 (0.992, 1.019) | 1.010 (0.987, 1.035) | 1.007 (0.991, 1.023) | 1.001 (0.987, 1.016) | 1.006 (0.988, 1.025) |
| No high school (%) | 0.993 (0.984, 1.003) | 0.985 (0.966, 1.004) | **0.987 (0.976, 0.998)** | 0.991 (0.981, 1.002) | 1.010 (0.997, 1.023) |
| Rent burden (%) | 1.001 (0.998, 1.005) | 1.005 (0.998, 1.011) | 1.002 (0.997, 1.006) | 1.000 (0.996, 1.003) | 1.001 (0.996, 1.005) |
| Under 18 (%) | **0.976 (0.959, 0.993)** | 0.993 (0.963, 1.025) | **0.971 (0.952, 0.991)** | **0.979 (0.961, 0.998)** | **0.960 (0.938, 0.983)** |
| Over 65 (%) | 1.002 (0.983, 1.021) | 1.002 (0.967, 1.038) | 0.985 (0.963, 1.007) | 0.995 (0.975, 1.016) | **1.029 (1.003, 1.056)** |
| Racial minority (%) | 1.001 (0.991, 1.010) | 1.000 (0.980, 1.021) | 0.995 (0.984, 1.006) | 0.997 (0.987, 1.008) | **1.018 (1.005, 1.032)** |
| Single parent household (%) | **1.016 (1.005, 1.027)** | 1.010 (0.991, 1.029) | **1.016 (1.004, 1.029)** | **1.012 (1.000, 1.024)** | **1.016 (1.002, 1.031)** |
| No vehicle (%) | 0.995 (0.983, 1.007) | 1.001 (0.979, 1.025) | 0.999 (0.986, 1.012) | 0.997 (0.984, 1.010) | **0.980 (0.965, 0.996)** |
| Disability (%) | 0.992 (0.970, 1.014) | 0.978 (0.934, 1.026) | 1.000 (0.974, 1.026) | 1.003 (0.979, 1.027) | 0.993 (0.963, 1.023) |
| Limited English (%) | **1.015 (1.003, 1.028)** | 1.012 (0.985, 1.039) | **1.025 (1.010, 1.040)** | **1.015 (1.002, 1.029)** | 0.994 (0.978, 1.011) |
